# Report of a Case of Giant Sarcoma

**Published:** 1903-02

**Authors:** Murdock C. Smith

**Affiliations:** Lynn, Mass.


### REPORT OF A CASE OF GIANT SARCOMA.[1]

BY MURDOCK C. SMITH, D.D.S., M.D., D.M.D., LYNN, MASS.

It is not often that a little girl of less than twelve years of age is presented to this Society twice.

Late in the fall of 1894 an alveolar abscess developed, and before it finally healed up she lost the greater part of the inferior maxillary, from the region of the cuspid to the articulation, also the germs of the two bicuspids and the first molar. The attention of this Society was called to the extensive loss and great deformity at that time, but you will see that there was but little deformity prior to the second operation.

February 13, 1902.—In consultation at the Union Hospital, Lynn, I found a little growth on the mucous membrane about a half-inch in diameter and about one-eighth inch in elevation; it looked like a small piece of liver, apparently coming from the periosteum, situated on the anterior surface between the central and lateral incisors, with those teeth noticeably separated. The history of the case is that it had only been noticed about two weeks, and the patient had felt no pain. The surgical staff were almost unanimous in pronouncing it a little granulation tissue from a slight necrosis of the alveolar process. The writer refrained from making a diagnosis, on the ground of the separation of the teeth. Under ether the growth was removed and the bone curetted. A few days later the pathologist reported giant-cell sarcoma.

March 12.—Took her to Dr. Whitney at the Massachusetts General Hospital. At that time there was a decided return of the growth; still the doctor did not think there was any great hurry for a second operation.

March 23.—It has been growing so rapidly that we have decided

---

[1] Read before the Massachusetts Dental Society, June 4, 1902.

to operate at once, which was done at the Union Hospital, assisted by Drs. Lougee, Haywood, Ryder, and Smith. The mucous membrane was opened high up on the lip and the knife carried into the deep tissue almost to the skin and dissected well down on the body of the jaw; on the lingual surface the knife was carried just below the gingival border, and with a periosteal scaler the soft tissue was dissected from the bone; then with a circular saw and the trephine the bone was easily removed, well down below the alveolar process; the bleeding was mostly capillary, and after the large vessels were secured soon dwindled down to an oozing. The bleeding surface was cauterized with five per cent. solution of pyrozone and the wound packed with iodoform gauze.

April 3.—Left dressing out; healed up nicely.

In this case I wish to call attention to the amount of bone that can be removed and show so little deformity.

As we send all specimens of growth that we remove from the mouth to the pathologist this was sent after the diagnosis was made, and the following is the pathologist's report.

"The specimen from the girl aged eleven years removed March 23 consisted of a portion of the left half of the lower jaw, contained four teeth, and covered an area of about one centimetre, in which was a soft, reddish new growth.

"*Microscopic examination* showed the growth to be composed of relatively large irregularly rounded and spindle cells with little intercellular substance, among which were cells of a larger size, and contained from one to ten or more nuclei.

"*Diagnosis.*—Giant-cell sarcoma, probably of periosteal origin.

(Signed) "Wm. F. Whitney, M.D."

